# Multimodal Benefits of Exercise in Patients With Multiple Sclerosis and COVID-19

**DOI:** 10.3389/fphys.2022.783251

**Published:** 2022-04-14

**Authors:** Omid Razi, Bakhtyar Tartibian, Ismail Laher, Karuppasamy Govindasamy, Nastaran Zamani, Silvia Rocha-Rodrigues, Katsuhiko Suzuki, Hassane Zouhal

**Affiliations:** ^1^ Department of Exercise Physiology, Faculty of Physical Education and Sport Sciences, Razi University, Kermanshah, Iran; ^2^ Department of Sports Injuries, Faculty of Physical Education and Sports Sciences, Allameh Tabataba’i University, Tehran, Iran; ^3^ Department of Anesthesiology, Pharmacology and Therapeutics, Faculty of Medicine, University of British Columbia, Vancouver, BC, Canada; ^4^ Department of Physical Education & Sports Science, SRM Institute of Science and Technology, Kattankulathur, India; ^5^ Department of Biology, Faculty of Science, Payame-Noor University, Tehran, Iran; ^6^ Escola Superior de Desporto e Lazer, Instituto Politécnico de Viana do Castelo, Viana do Castelo, Portugal; ^7^ Research Centre in Sports Sciences, Health Sciences and Human Development (CIDESD), Quinta de Prados, Edifício Ciências de Desporto, Vila Real, Portugal; ^8^ Tumor & Microenvironment Interactions Group, i3S, Porto, Portugal; ^9^ Faculty of Sport Sciences, Waseda University, Tokorozawa, Japan; ^10^ Laboratoire Mouvement, Sport, Santé, University of Rennes, Rennes, France; ^11^ Institut International des Sciences du Sport (2I2S), Irodouer, France

**Keywords:** COVID-19, multiple sclerosis, blood-brain barrier, glia, physical exercise, myelin

## Abstract

Multiple sclerosis (MS) is a demyelinating disease characterized by plaque formation and neuroinflammation. The plaques can present in various locations, causing a variety of clinical symptoms in patients with MS. Coronavirus disease-2019 (COVID-19) is also associated with systemic inflammation and a cytokine storm which can cause plaque formation in several areas of the brain. These concurring events could exacerbate the disease burden of MS. We review the neuro-invasive properties of SARS-CoV-2 and the possible pathways for the entry of the virus into the central nervous system (CNS). Complications due to this viral infection are similar to those occurring in patients with MS. Conditions related to MS which make patients more susceptible to viral infection include inflammatory status, blood-brain barrier (BBB) permeability, function of CNS cells, and plaque formation. There are also psychoneurological and mood disorders associated with both MS and COVID-19 infections. Finally, we discuss the effects of exercise on peripheral and central inflammation, BBB integrity, glia and neural cells, and remyelination. We conclude that moderate exercise training prior or after infection with SARS-CoV-2 can produce health benefits in patients with MS patients, including reduced mortality and improved physical and mental health of patients with MS.

## Introduction

The coronavirus disease 2019 (COVID-19) pandemic that started in 2020 has caused death and disease throughout the world (Morley, 2020), leading to emergency measures such as extended locking-downs of communities, public places, stay at home requirements (Faulkner et al., 2020), and a disruption of daily life, leading to social insulation, and loneness (Berg-Weger and Morley, 2020). The SARS-CoV-2 virus can dissipate to the central nervous system (CNS) and culminates in delirium, depression and other mental, and psychological disorders due to the increased levels of inflammatory cytokines in the brain (Perez et al., 2020). SARS-CoV-2 is a neuroinvasive virus that not only triggers circulatory inflammation, but also affects various components of the CNS (Mao et al., 2020b; LiY-C and Hashikawa, 2020). Infection with COVID-19 leads to a disruption of the blood-brain barrier (BBB) disruption, increased reactivity of astrocytes (astrogliosis), microgliosis, myelin damage and demyelination, and neuronal loss and the formation of plaques (Gowrisankar and Clark, 2016; Ahmadirad and Ghasemi, 2020; Li L. et al., 2020; Calcagno et al., 2020; Desforges et al., 2020; Nordvig et al., 2021; Tavčar et al., 2021). The burden of infection with COVID-19 varies in different individuals, such that some individuals experience no signs of infection while others who suffer autoimmunity and inflammatory diseases such as multiple sclerosis (MS) may experience more intense symptoms of infection (Zindler and Zipp, 2010; Wilson et al., 2020).

Many features of MS and COVID-19 disease share similarities, including immune and BBB dysfunction, increased reactivity of brain residence cells (astrogliosis, microgliosis), demyelination and plaque formation, and finally neuronal loss (Thompson, 2000; Sofroniew, 2009; Brosnan, 2013; Rempe et al., 2016; Troletti et al., 2016). Inflammation in both diseases is associated with plaque formation (Minagar et al., 2002; Minagar and Alexander, 2003; Nordvig et al., 2021). The formation of plaques/lesions leads to neurological symptoms that result from neuronal loss and oxidative stress (Gilgun-Sherki et al., 2004; Kostic et al., 2013). Many patients with MS disease become less active and this inactivity leads to an accumulation of adipose tissue. Accrued adipose tissue triggers inflammatory conditions and plaque formation (Elenkov et al., 2005; Maes et al., 2009). Concurrent exposure of MS patients with the hyper-inflammation associated with coronavirus infection exposes individuals with MS at a higher risk of psychological issues and a more extensive neuropathology (Miller et al., 2009; Iwata et al., 2013; Rethorst et al., 2013; Gasmi et al., 2020).

The high transmissibility and dissemination of the coronavirus has led to a race to develop treatments for COVID-19 using inactivated/killed whole virus and convalescent plasma to improve immune responses (See et al., 2008; Dai and Gao, 2021). These vaccines can cause liver pathology and a robust response of the immune system especially in T cells and antibody titers in individuals with COVID-19 (Weingartl et al., 2004; Zhao et al., 2005; Shi et al., 2006; Ip et al., 2014; Al-Amri et al., 2017). Despite these complications, many patients with MS and who are infected with COVID-19 will receive vaccinations against COVID-19. It is not clear that how long the benefits of the COVID-19 vaccines will provide protection. Lifestyle changes, as proposed by the World Health Organization (WHO) (World Health Organization, 2004), involving increased daily physical activity is an alternate non-pharmacological procedure in the management of COVID-19 where engaging in physical activity or physical therapy could inhibit COVID-19 transmission (Sang et al., 2020) and also improve psychological, neurological, and physical health (Chekroud et al., 2018; Mücke et al., 2018; Schuch and Stubbs, 2019).

There is no molecular-based evidence regarding the effects of COVID-19 in individuals with MS. The goal of this review is to describe the effects of the coronavirus pandemic on patients with MS and the neuroprotective roles of exercise.

## Literature Search Strategy

A comprehensive revision was performed using electronic databases including Medline, ISI Web of Knowledge, PubMed, Google Scholar, and Scopus on studies related to human and experimental subjects, from inception until February 2022. We included studies involved MS, COVID-19, and exercise and investigations on mechanisms, using the following key terms: “coronavirus or COVID-19”, “MS disease or patients”, “coronavirus and nervous system”, “coronavirus pathways infecting central nervous system”, “coronavirus and cytokine storm”, “coronavirus and BBB disruption”, “coronavirus and microglia activation”, “coronavirus and astrocyte activation”, “microglia and astrocytes in health and pathology”, “coronavirus/MS and demyelination”, “coronavirus and plaque/lesion formation”, “coronavirus and neural loss”, “MS and cytokine storm”, “MS and inflammation”, “MS and BBB permeability”, “MS and plaque/lesion formation”, “COVID-19/MS and mental or psychological or mood or anxiety or depression problems”, “COVID-19 and loneliness”, “COVID-19 and stress”, “loneliness and stress”, “COVID-19 and socio-psychological stress”, “COVID-19/MS physical inactivity”, “physical inactivity and mental/psychological/mood problems”, “physical inactivity and inflammation”, “inflammation and depression”, “exercise adaptions in mental/mood/psychological/metabolic/central nervous system”, “exercise and the changes in myokines”, “exercise and immune system or inflammation”, “exercise and monoamines and neurotransmitters”, “exercise and BBB”, “exercise and microglial/astrocytes changes”, “exercise and neurotrophic/growth factors”, “neurotrophic/growth factors and depression”, “exercise and endocrine adaptations”, “exercise and neurological/mental/mood disorders”, “exercise and stress reduction”, “exercise and endocannabinoids”, “endocannabinoids and mood”, “monoamines and psychological problems”, “exercise and oxidative stress”, “exercise and MS”, “exercise during COVID-19”, “exercise and opioids”, “opioids and mood”, “exercise and stress”.

## Coronavirus as a Neuro-Invasive Virus

The worldwide pandemic caused by COVID-19 led to a novel disease related to severe acute respiratory syndrome coronavirus-2 infection (SARS-CoV-2) (Zhu N. et al., 2020; Zhou et al., 2020). SARS-CoV-2 is beta-coronavirus that is closely associated with the SARS-CoV virus dissipated in 2002–2004 (Zhou et al., 2020). Human coronaviruses (HCoVs) were initially categorized into seven strains 1) SARS-CoV, 2) Middle East Respiratory Syndrome-related Coronavirus (MERS-CoV), 3) HCoV-229E, 4) HCoV-OC43, 5) HCoV-NL63, 6) HCoV-HKU1, and 7) the novel SARS-CoV-2 (Bohmwald et al., 2018). The genome of HCoVs encodes proteins and glycoproteins such as the spike glycoprotein, membrane glycoprotein, envelope glycoprotein, nucleocapsid protein, RNA polymerase and some genes for accessory proteins (Cheng Q. et al., 2020). Three (MERS-CoV, SARS-CoV, and SARS-CoV-2) out of the seven HCoVs cause severe respiratory illness with high incidence and mortality rates (Cheng Q. et al., 2020). SARS-CoV-2 shares 79 and 50% genomic similarities to SARS-CoV and MERS-CoV, respectively (Lu et al., 2020). The respiratory tract is the main target tissue for HCoVs (Abdelaziz and Waffa, 2020), though 5% of patients that need ventilatory support (Day, 2020) have a 40–50% mortality rate (Zhu J. et al., 2020; Weiss and Murdoch, 2020). Mortality rates due to COVID-19 are high in the elderly, likely due to the presence of comorbidities such as cardiovascular diseases, smoking, lung disease, obesity and diabetes (Zhu N. et al., 2020; Richardson et al., 2020), although fatal outcomes can also occur in otherwise healthy younger patients but with high viral loads (Chen et al., 2020). Common clinical features associated with infection with COVID-19 include fever, sore throat, dry cough, shortness of breath, and sometimes reproductive system dysfunction (Zhu J. et al., 2020; Richardson et al., 2020; Maleki and Tartibian, 2021).

SARS-CoV-2 infects neuronal cells of the CNS and peripheral nervous system (Mao et al., 2020b; Li et al., 2020c). Animal and post-mortem analysis indicates the thalamus, brainstem, cerebrum, hypothalamus, and cortex are the most infected areas (Gu et al., 2005; McCray et al., 2007; Netland et al., 2008). Neurological presentations such as headache, impaired consciousness, cognitive deficits, dizziness, acute ischemic stroke, intracerebral hemorrhage, nausea, and vomiting, anosmia, hypogeusia are frequent signs of neurovirulent infections with SARS-CoV-2 (Li et al., 2020b; Mao et al., 2020b). Brain edema and partial neurodegeneration have been identified in autopsies of COVID-19 patients (Ahmadirad and Ghasemi, 2020). These neurological signs occur in 88% patients with severe infections of COVID-19 (Mao et al., 2020b), while other reports suggest that one third of patients have neurological symptoms 2–3 weeks after infection with the virus (Mao et al., 2020a; Mao et al., 2020b). The brain is affected by SARS-CoV-2 infection after changes are observed in the respiratory tract, although this is not always the case (Filatov et al., 2020).

SARS-CoV-2 binds to angiotensin-converting enzyme 2 (ACE2) receptors that are primarily expressed on airway epithelial cells, lung parenchyma, vascular endothelial cells, renal cells, and cells in the small intestine (Kuhn et al., 2004; Lu et al., 2013; Qian et al., 2013; Zhu Y. et al., 2020), although this receptor is not expressed in brain tissue (Wei et al., 2020). The presence of ACE2 receptors is not sufficient to predispose host cells to CoV-induced infection (Gandhi et al., 2020). There are several mechanisms by which SARS-CoV-2 virus can enter the CNS and consequently damage neurons, including the trans-neuronal route which infects sensory and motor nerve terminals, especially the olfactory nerves in the nasal cavity, and subsequent entry to the CNS through retrograde transportation via motor proteins (Swanson and Mcgavern, 2015). The virus can also access respiratory and cardiovascular centers in the brainstem during the early stages of the disease and lead to respiratory failure, inflammation and demyelination reactions through plaque formation (Netland et al., 2008; Mori, 2015; Desforges et al., 2020; Steardo et al., 2020). The neuroinvasive nature of infection with COVID-19 was shown in intranasal swabs from mice infected with SARS-CoV where the virus reached the thalamus and brainstem areas via olfactory nerves (Kumari et al., 2021). The anosmia frequently reported by patients infected with COVID-19 provides additional evidence for an important role for viral entry into the CNS via olfactory nerves (Gandhi et al., 2020).

A second pathway for the entry of the virus is the hematogenous pathway which allows for viral entry of most HCoV strains by infecting peripheral monocytes, T lymphocytes, and macrophages (Wan et al., 2021). The infected immune cells can cross the BBB of the ventricular choroid plexus through transcytosis (Gu et al., 2005; Chan et al., 2013; Desforges et al., 2014; Calcagno et al., 2020). A third pathway for viral entry into the CNS involves the microvascular endothelial cells of the BBB (Ahmadirad and Ghasemi, 2020). Endothelial cells of the BBB express receptors for SARS-CoV such as ACE2 and CD209L (Li et al., 2007). Glial cells and neurons in the brainstem, sub-fornical organ, paraventricular nucleus, nucleus tractus solitarius, rostral ventrolateral medulla potentially also express ACE2 receptor for SARS-CoV-2 (Xia and Lazartigues, 2010; Gowrisankar and Clark, 2016). Viral spike glycoproteins interact with ACE2 receptors on the surface of capillary endothelial cells to disrupt the integrity of the BBB (Baig et al., 2020). Infection with the SARS-CoV-2 increases the permeability of the BBB as a result of a cytokine storm due to the direct cytopathic effects of the virus (Calcagno et al., 2020; Nordvig et al., 2021), suggesting that ACE inhibitors could be a treatment option in some infected patients with hypertension and/or diabetes (Yan et al., 2020). The interaction of SARS-CoV-2 with ACE2 receptors on vascular endothelial cells can increase blood pressure, disrupt the BBB or lead to intracerebral hemorrhage (Calcagno et al., 2020). Stimulation of the immune system by SARS-CoV-2 can compromise BBB integrity through two main mechanisms: downregulation of tight-junction proteins (Desforges et al., 2020) and activation of resident glial cells, particularly astrocytes, in the CNS (Arbour et al., 2000).

**FIGURE 1:**
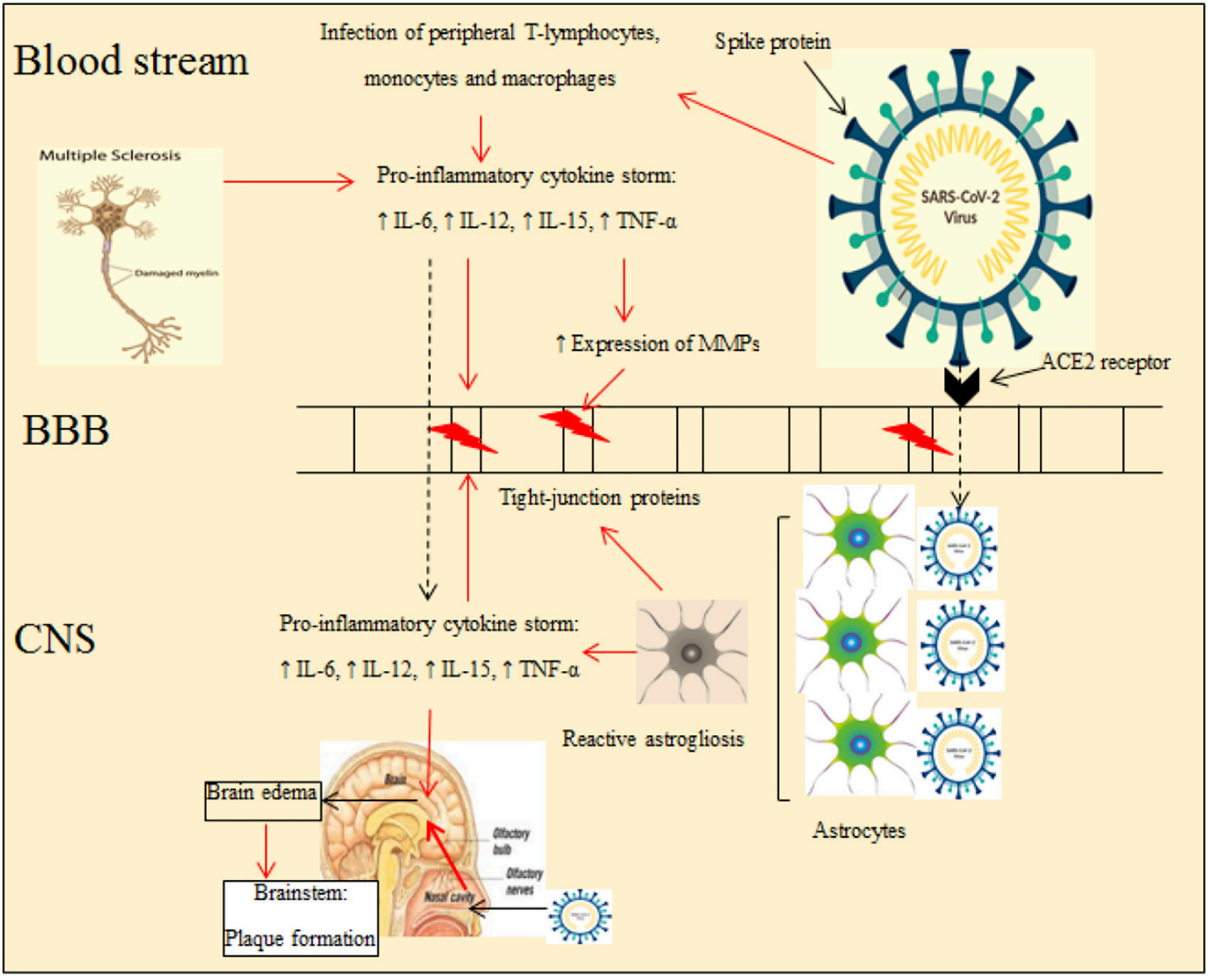
Neuro-invasive mechanisms of SARS-CoV-2 and common effects of COVID-19 and MS diseases on brain and its components. Hyper-inflammation induced by MS and coronavirus increases BBB permeability and plaque formation in the brainstem. Reactivation of astrocytes induced by pro-inflammatory cytokines also impacts the BBB. The red arrows indicate the detrimental effects of inflammation produced by both MS and SARS-CoV- 2 (ACE2, angiotensin-converting enzyme 2; MMPs, matrix metalloproteinases; MS, multiple sclerosis; CNS, central nervous system; BBB, blood-brain barrier).

**FIGURE 2:**
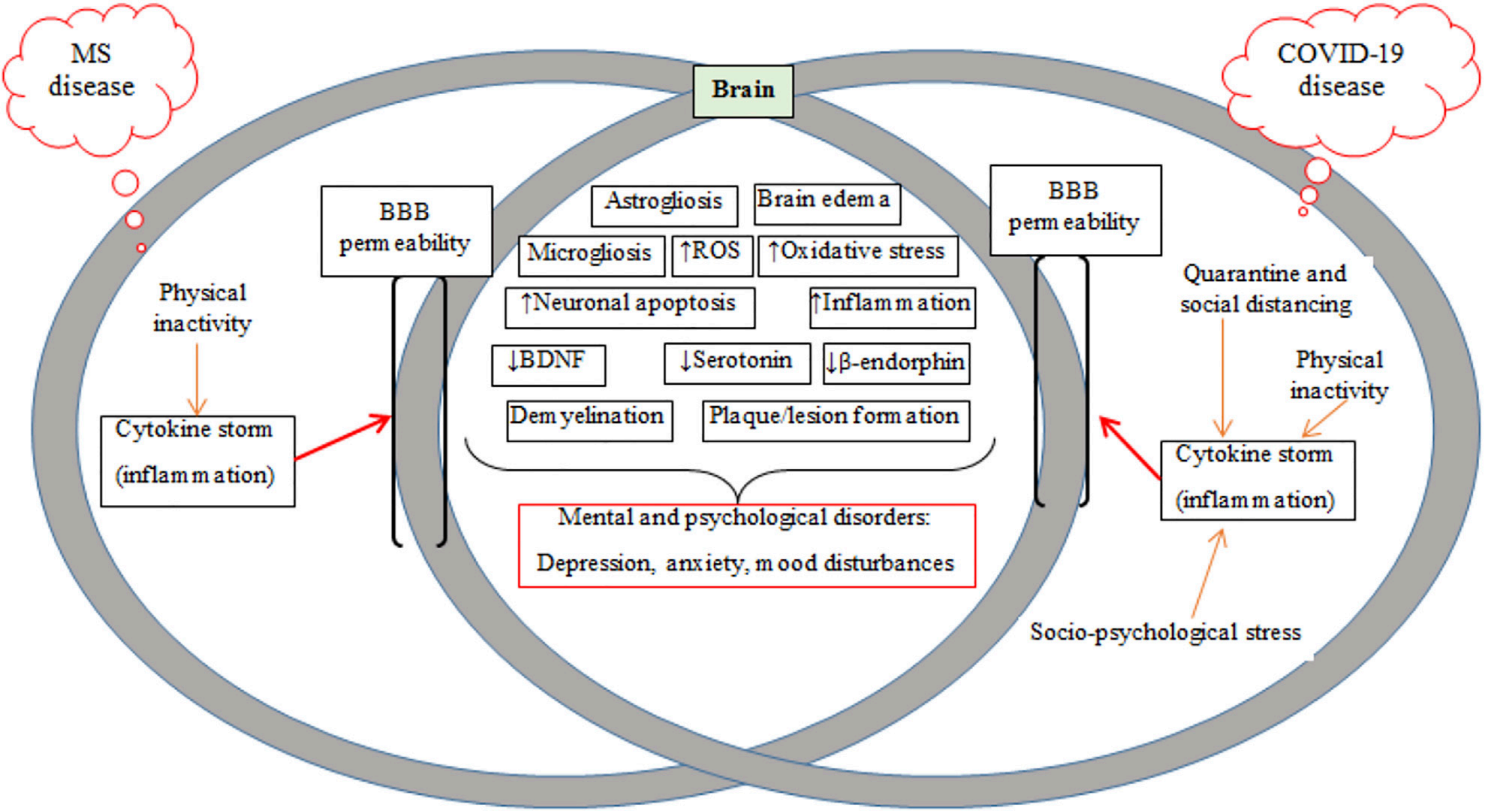
Schematic of mechanisms common in MS and COVID-19 diseases, including BBB breakdown/permeability and mental and psychological disorders. MS, multiple sclerosis; BBB, blood-brain barrier; ROS, reactive oxygen species; BDNF, brain-derived neurotrophic factor.

Peripheral T lymphocytes, neutrophils, natural killer cells, and monocyte/macrophages secrete matrix metalloproteinases (MMPs) to increase BBB permeability by downregulating tight junction proteins such as occludin and cluadin-5 (Nordvig et al., 2021). Hyper-inflammation induced by SARS-CoV-2 stimulates astrocytes (Calcagno et al., 2020), a highly heterogeneous group of cells with plasticity in form and function. Astrocytes regulate homeostasis in the CNS by transporting ions and protons, eliminating and catabolizing neurotransmitters, scavenging reactive oxygen species (ROS) and providing neurons with energy substrates. Astrocytes maintain the cytoarchitecture by connecting their end-feet to the vasculature to regulate BBB permeability (Verkhratsky and Nedergaard, 2018; Verkhratsky and Zorec, 2019). Astrocytes are targeted by CoVs through binding to ACE2 and Toll-like receptors expressed on astroglial cells (Calcagno et al., 2020; Li et al., 2020a). The interaction of astrocytes and microglia with pathogens such as SARS-CoV-2 leads to reactive astrogliosis and microgliosis (Figures 1, 2) (Chen et al., 2021; Tavčar et al., 2021). Reactive astrogliosis is defined by changes morphology (hypertrophy) and increased gene expression, such as glial fibrillary acidic protein (GFAP) (Ben Haim et al., 2015), which modulates the stability of astrocytes and the severity of brain injury (Trautz et al., 2019). Morphological alterations of these glial cells are congruent with the loss of physical connections between reactive astrocyte and endothelial cells and are associated with increased BBB permeability (Chupel et al., 2018). Additionally, astrogliosis increases the production of pro-inflammatory cytokines, chemokines and other inflammatory signals that disrupt the BBB and consequently promotes neuronal inflammation (Wang et al., 2020). Patients infected with COVID-19 experience damage to both white and gray matter and have demyelinating lesions (plaques) throughout the brain, including the cerebellum, brainstem, and spinal cord (Arbour et al., 2000; Yeh et al., 2004; Algahtani et al., 2016; Li et al., 2016; Morfopoulou et al., 2016) (Figure 1).

Collectively, SARS-CoV-2 is a neuroinvasive virus that enters the CNS and leads to inflammation, changes in glial cells BBB permeability, plaque formation and demyelination (Yachou et al., 2020).

## Susceptibility of Patients With MS to Coronavirus Infection

Disruption of the BBB leads to inflammation in both patients with MS and in experimental autoimmune encephalomyelitis (EAE), an animal model of MS (Brosnan, 2013; Troletti et al., 2016; Razi et al., 2022a). Several agents modulate BBB permeability in MS/EAE, including adhesion molecules, ROS, pro-inflammatory cytokines such as IL-1β, interferon-gamma (IFN-ϒ), and tumor necrosis factor-alpha (TNF-α) (Abbott et al., 2006; Alvarez et al., 2011; Baeten and Akassoglou, 2011). Partial dysregulation of tight junction proteins are mediated by inflammatory immune mechanisms and the activation of MMPs by resident activated glial cells and pro-inflammatory immune cells (Rempe et al., 2016). Additionally, astrocytes are the first glial cells to respond to immune insults (Sofroniew, 2009).

Plaque/lesion progression is a hallmark of MS pathology. Oligodendrocytes, as glial cells forming myelin sheaths around axons involved in conducting neuronal impulses, are targeted by the immune system in acute lesions in patients with MS (Thompson, 2000). Although the origin of the disease is unknown, it is assumed that there is an interaction between receptors on T cells with myelin antigens present on major histocompatibility complex (MHC) class II molecules, which are expressed on macrophages/microglia and astrocytes (Minagar and Alexander, 2003). Activated lymphocytes and macrophages cross the BBB and interact with resident cells in the CNS such as microglia and astrocytes (Smith et al., 1998) (Figure 1). Pro-inflammatory cytokines activate microglia and increase quinolinic acid levels in the CNS and also stimulate apoptosis (Figure 2) (Myint et al., 2007; McNally et al., 2008). Astroglial loss in MS patients has several detrimental consequences on brain health, including decreased re-uptake of glutamate from the synaptic cleft to induce neuronal excitotoxicity, decreased metabolic support via lactate production, and increased oxidative stress through the loss of astrocyte produced antioxidants (Phillips and Fahimi, 2018). Additionally, quinolinic acid acts on ionotropic N-methyl-D-aspartate receptor (NMDA) and glutamate receptors to inhibit the upregulation of neurotrophic factor like brain-derived neurotrophic factor (BDNF) mediated by cAMP response element-binding protein (CREB) (Hardingham et al., 2002; Santana-Martínez et al., 2019). Inflammatory mediators such as pro-inflammatory cytokines (TNF-α, IFN-ϒ) and chemokines are released by proliferating lymphocytes (Fujiwara and Kobayashi, 2005). The release of these mediators leads to an imbalance between pro- and anti-inflammatory (IL-4 and IL-10) cytokines in favor of inflammatory mechanisms (Minagar and Alexander, 2003; Raivich and Banati, 2004). Thus, MS is likely also associated with cytokine storms (Link, 1998). Myelin is injured by toxic substances such as oxygen and nitrogen free radicals produced by an auto-activated immune system and myelin then is phagocytized by macrophages and microglia (Smith et al., 1998; Rus et al., 2006). Injured myelin disrupts the axonal conduction and gradually leads to axonal loss and lesion or plaque formation (Van Asseldonk et al., 2003; Waxman, 2006). Patients in the early phases of MS experience functional disorders derived from produced hyper-inflammation (Figure 2) (Minagar et al., 2002; Minagar and Alexander, 2003), but the clinical symptoms observed later derive from degenerative changes or lesions (Chang et al., 2002).

The formation of lesions or plaques is a hallmark in the pathology of MS, with imaging of lesions confirmed by MRI technology used to diagnose the severity of MS (Ge, 2006). With respect to the diagnosis of relapsing-remitting (RR) or progressive MS, the lesions in white and gray matter have been categorized into two general groups: inflammatory and degenerative lesions (although they are sometimes also classified as acute and chronic lesions) that are heterogeneous in size and shape (Barkhof et al., 1992). Collectively, the lesions are distributed in most regions of the brain such as periventricular and sub-cortical regions, corpus callosum, brainstem, and optic nerves (Paolillo et al., 1997). These underlying inflammatory and demyelinating conditions can predispose patients with MS to more severe neurological changes and challenges following COVID-19 infection, as this virus causes symptoms that are also present in MS (Naucler et al., 1996; Ahlqvist et al., 2005; Donati et al., 2005). Inhibiting the migration of oligodendrocyte precursor cells to demyelinated sites by SARS-CoV-2 may be another mechanism for exacerbating neuronal damage in MS following infection with COVID-19 (Kong et al., 2003; Campbell et al., 2017). Some currently used vaccines could be harmful in some patients with autoimmunity such as MS, since the most of these vaccines contain the S1 subunit of spike protein of the SARS-CoV-2 in an inactivated form (Dai and Gao, 2020; Ong et al., 2020; Biström et al., 2021), which can activate systemic and central inflammation (Rhea et al., 2021). Importantly, SARS-CoV-2 can cause MS plaques in the brainstem to extend to other regions controlling the respiratory system (Berger et al., 2020; Li et al., 2020b).

## The Concurrent Effects of COVID-19 and MS on Mental and Psychological Factors

Common strategies to combat viral infections include quarantine and social isolation, which can cause mental and psychological disorders including acute and chronic stress, anxiety and depression (Garcovich et al., 2020; Hwang et al., 2020; Pan et al., 2020; Wilkialis et al., 2021). These countermeasures are associated with sedentary behaviors that decrease physical exercise (Booth et al., 2000; Lightfoot, 2011; Gasmi et al., 2020). Neurological diseases such as MS also decrease physical activity (Lee et al., 2003; Barnes and Yaffe, 2011). Physical inactivity and socio-psychological stress lead to inflammatory conditions with oxidative damage to lipids, proteins and DNA in the brain (Liu et al., 1996; Radak et al., 2001). Chronic stress associated with the coronavirus pandemic produces physiological changes such as increased release of cytokines, cortisol and catecholamines, and can lead to depression (Raison and Miller, 2003; Iwata et al., 2013; Mariotti, 2015; Dos Santos, 2020; Ramezani et al., 2020; Mohammadkhanizadeh and Nikbakht, 2021).

As many as 60% of patients with MS suffer with depression and anxiety (Bakshi et al., 2000; Grytten et al., 2016). There are several mechanisms that can culminate in depression in patients with MS and SARS-CoV-2 infection (Machado and Gutierrez, 2020). The stress response is largely regulated by the hypothalamic-pituitary-adrenal (HPA) axis (which releases cortisol) and the sympathetic nervous system (which releases epinephrine and norepinephrine) (Silverman and Deuster, 2014). The initial response to stress is characterized with increases in catecholamine secretion and is associated with the trafficking leukocytes from the spleen to the circulation (Silverman and Deuster, 2014). Increased cortisol levels induced by chronic stress reduces immune function (McEwen et al., 1997; Sapolsky et al., 2000; Dhabhar, 2009), increasing the susceptibility of patients with MS to infection (Chaudhry et al., 2021; Zabalza et al., 2021). High levels of cortisol and inflammatory cytokines in both MS and COVID-19 downregulate the protective effects of BDNF against neuronal cell apoptosis (Zhao et al., 2007; Kudielka et al., 2009; Tollenaar et al., 2009). Peripheral and central BDNF levels are reduced in MS (Connor et al., 1997; Baker et al., 2010; Laske et al., 2010; Pereira et al., 2013), and are associated with signs of depression and anxiety due to reduced release of synaptic proteins and neurotransmitters (monoamines and opioids) (Egan et al., 2003; Hariri et al., 2003; Vaynman et al., 2006). Increased neuronal apoptosis in the brain causes neuronal loss and reduces the number of synapses (Glantz et al., 2006; Zhou et al., 2019). Neuronal excitotoxicity produced by high glutamate concentrations in patients with MS could be another mechanism to downregulate BDNF by inhibiting CREB binding to DNA (Zou and Crews, 2006; Kostic et al., 2013). Oxidative stress is important in the pathogenesis of neuro-inflammatory diseases such as MS (LeVine, 1992; Sayre et al., 2008). Although multiple factors contribute in oxidative stress, including glutamate induced activation of ionotropic receptors (Gilgun-Sherki et al., 2004). Oxidative stress causes damage to DNA that further increases apoptosis and neurodegeneration (Bohr et al., 1998; Schmitz et al., 1999).

Inflammation and depression is common in patients with MS and those infected with COVID-19, (Figure 2) (Möller et al., 2013; Phillips and Fahimi, 2018). Increased pro-inflammatory markers [IL-6, IL-1β, TNF-α, and C-reactive protein (CRP)] occur in individuals with depression (Laske et al., 2008; Steiner et al., 2012), supported by reports of anti-depressant effects of TNF-α antagonists (Soczynska et al., 2009; Fond et al., 2014; Abbott et al., 2015). Pro-inflammatory cytokines activate the kynurenine pathway to form quinolinic acid or kynorenic acid; quinolinic acid activates NMDA receptors and inhibits the upregulation of *Bdnf* gene by blocking CREB function (Hardingham et al., 2002). Downregulation of BDNF expression reduces neurogenesis and neurotransmission (Galic et al., 2012; Calabrese et al., 2014).

Pro-inflammatory cytokines activate microglial cells in the CNS (McCusker and Kelley, 2013), which then intensifies inflammatory activation by secreting other pro-inflammatory cytokines such as IL-1β, TNF-α, IL-6, and IFN-γ (Smith et al., 2012). This excessive production of pro-inflammatory cytokines increases the firing rates of adrenergic neurons that inhibit beta-endorphin neurons (Rivier, 1995; Boyadjieva et al., 1997; Borsody and Weiss, 2002). This can lead to mood changes in MS patients with COVID-19 infection as mood is regulated by endorphin levels (Figure 2) (Fichna et al., 2007). Two other mechanisms whereby patients with MS and infected with COVID-19 can experience alterations in mood, depression and anxiety are increased activation of inhibitory GABAergic neurons that reduces firing rate of serotonergic neurons (Manfridi et al., 2003; Brambilla et al., 2007), and reduced activation of serotonin transporters that lower serotonin levels in nerve terminals (Haase and Brown, 2015). Importantly, central inflammation can cause neural apoptosis in some brain areas regulating these psychological states is also related to mitochondrial dysfunction (Cotman and Berchtold, 2002; Butterfield et al., 2014; Kiliaan et al., 2014).

In summary, increased levels of pro-inflammatory cytokines change neuroendocrine function, neurotransmitter metabolism and neuroplasticity to cause detrimental effects on psychological states (Elenkov et al., 2005; Maes et al., 2009; Miller et al., 2009; Rethorst et al., 2013). Individuals with clinical depression and anxiety disorders experience changes in mood, energy loss, and reduced exercise levels (Dinas et al., 2011).

## Exercise Training in MS and Covid Infection

**FIGURE 3:**
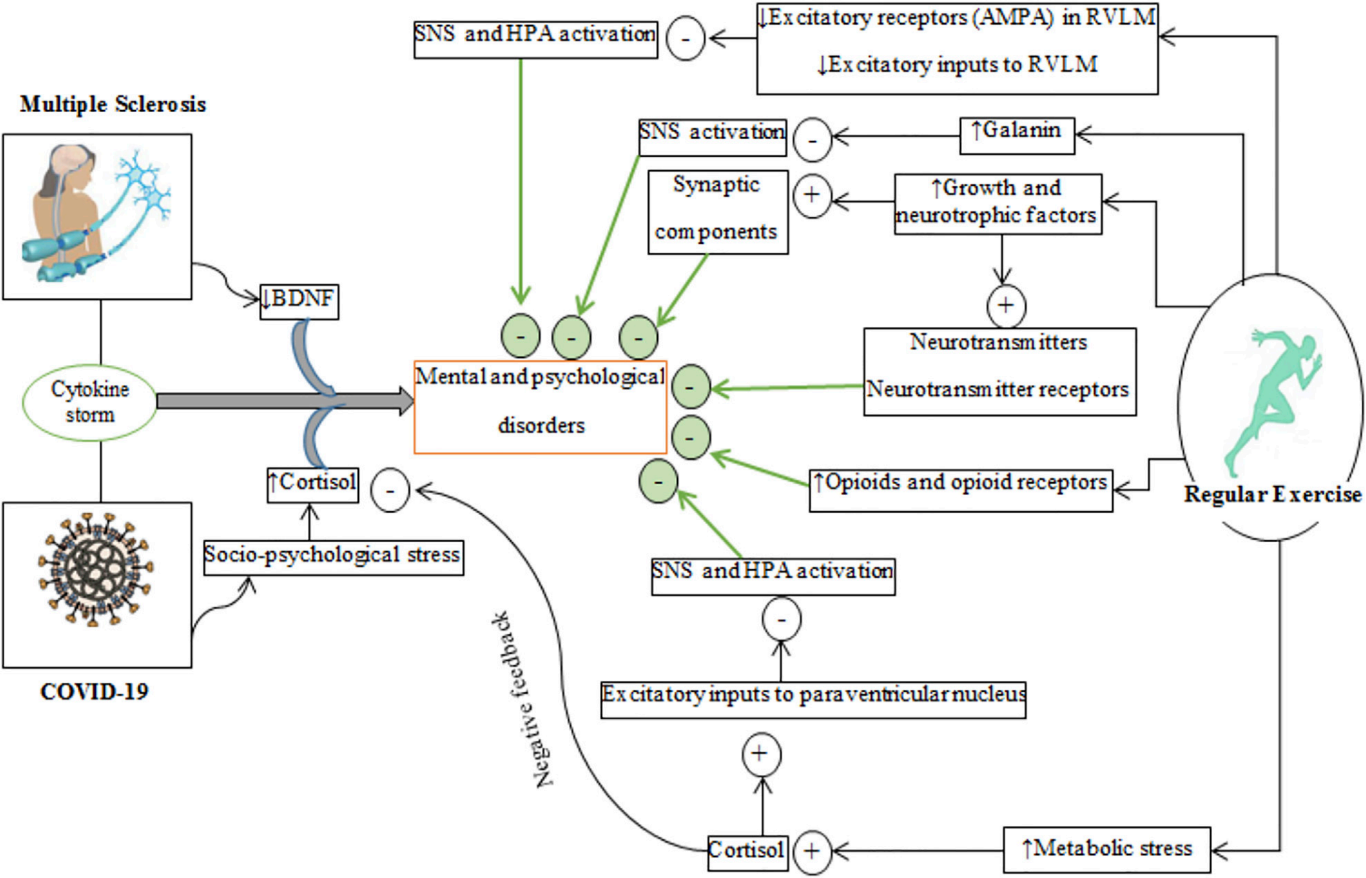
Schematic representation of converged pathways associated with mental and psychological disorders at (left side) and regular exercise that inhibit these disorders (right side) in multiple sclerosis and COVID-19. Plus (+) marks represent the incremental effects of exercise while minus (−) marks represent decreased effects of exercise. The effects of regular exercise are shown by thick green arrows. HPA, hypothalamus-pituitary-adrenal axis; SNS, sympathetic nervous system; BDNF, brain-derived neurotrophic factor; AMPA, α-amino-3-hydroxy-5-methyl-4-isoxazolepropionic acid; RVLM, rostral ventrolateral medulla.

**FIGURE 4:**
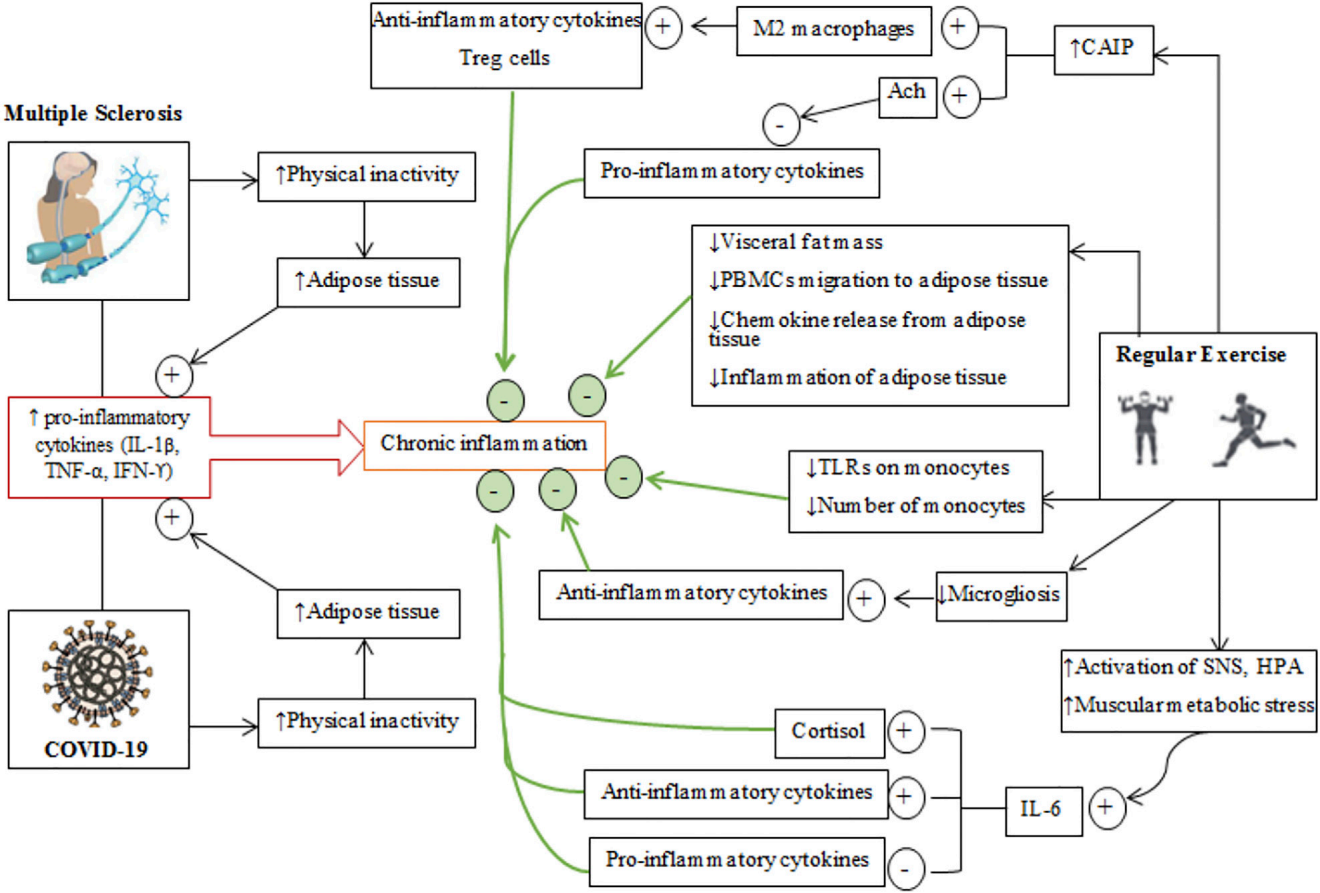
Schematic depiction of the effects of regular exercise on inflammatory conditions in multiple sclerosis and COVID-19 diseases. The generally common and different pathways through which MS and COVID-19 cause an inflammatory condition are shown on the left while isolated effects of regular exercise that regulate/modulate inflammation are shown on the right. Plus (+) signs represent the incremental effects of exercise, while minus (−) signs represent decreased effects of exercise. The cumulative effects of regular exercise are shown by the thick green arrows and green circles. Treg cells, T regulatory cells; PBMCs, peripheral blood mononuclear cell; CAIP, cholinergic anti-inflammatory pathway; SNS, sympathetic nervous system; ACh, acetyl choline; TLRs, toll-like receptors.

**FIGURE 5:**
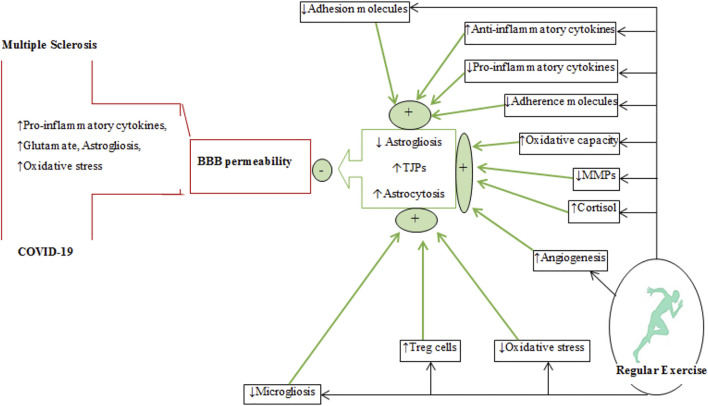
A schematic diagram depicting the effects regular exercise on BBB permeability in multiple sclerosis and COVID-19. The plus (+) and minus (−) signs represent increases and decreases, respectively. The effects of regular exercise are shown by thick green arrows. MMPs, matrix metalloproteinases; Treg, T regulatory cells; TJPs, tight-junction proteins; BBB, blood-brain barrier.

**FIGURE 6:**
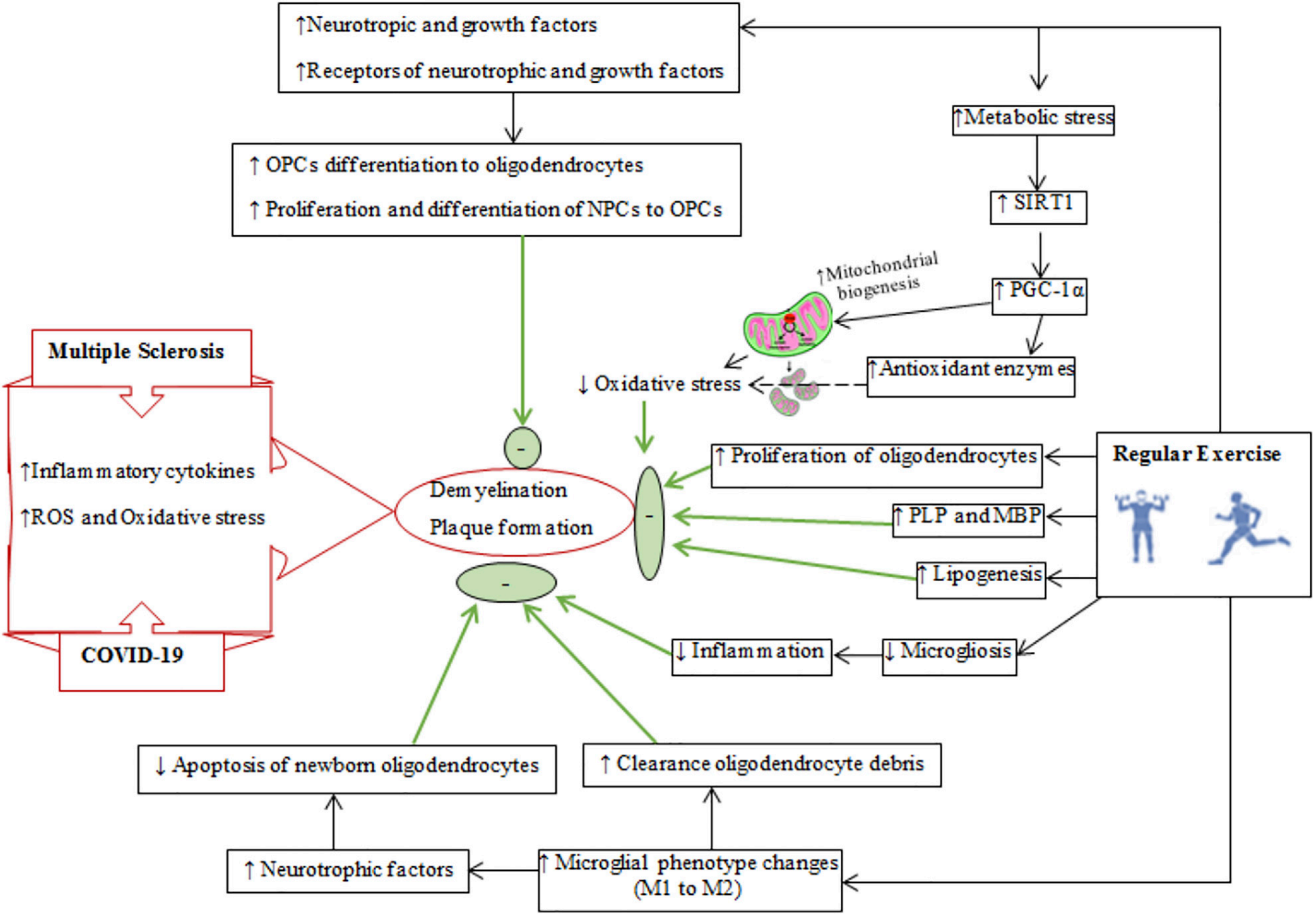
Schematic diagram of demyelination and plaque formation in multiple sclerosis and COVID-19. The reparative effects of regular exercise on reducing demyelination and improving remyelination are shown on right side. The final effects of regular exercise are shown by thick green arrows and green circles and oval shapes. SIRT1, silent mating type information training; PGC-1α, peroxisome proliferator-activated receptor-gamma coactivator-1 alpha; PLP, myelin proteolipid protein; MBP, myelin basic protein; NPCs, neuronal precursor cells; OPCs, oligodendrocyte precursor cells.

Regular physical exercise improves cardiovascular/aerobic capacity and brain health (Colcombe and Kramer, 2003; Etnier et al., 2006). Exercise causes adaptations to organs such as the liver, skeletal muscles, adipose tissue, and brain at molecular and cellular levels (Gomez Pinilla, 2006; Rasmussen et al., 2009; Di Liegro et al., 2019). Exercise exerts its beneficial effects on the brain by producing neurotrophic, growth and myokine factors and also by altering neurotransmission, improving BBB integrity, increasing remyelination and improving the immune system (refer to Figures 3–6) (Lin and Kuo, 2013; Phillips et al., 2014).

## Exercise Training and Mental and Psychological Health

Exercise is often recommended in the management of chronic diseases such as neurological, endocrine, psychological, and mental diseases (Weyerer and Kupfer, 1994; Brandt and Pedersen, 2010; Carek et al., 2011; Pedersen and Saltin, 2015; Baek, 2016; Spielman et al., 2016; Smith and Merwin, 2021). The beneficial effects of exercise on mental health markers involve reduced stress, anxiety and depression (Figure 3), increased neurotrophic and growth factors, increased neurotransmitters and synaptogenesis, elevated endogenous opioid levels markers, and changes in the expression and affinity of some central monoamine receptors (Figure 3) (Lin and Kuo, 2013; Phillips and Fahimi, 2018).

Trained males and females have lower responses of cortisol and heart rate related to socio-psychological stress (Rimmele et al., 2009), and as a consequence have lower anxiety and improved mood when encountering stressful conditions such as the COVID-19 pandemic (Silverman and Deuster, 2014). Physical exercise in sedentary individuals generally reduces HPA and sympathetic nervous system activity (Zschucke et al., 2015), which can improve lower stress levels such as during the COVID-19 pandemic (Figure 3) (Luger et al., 1988; Deuster et al., 1989), primarily by: 1) secreting cortisol due to metabolic exercise stress (Webb et al., 2013) added to socio-psychological stress (e.g., induced by COVID-19) for igniting negative feedback which negatively regulates hypothalamus and pituitary glands (Basso and Suzuki, 2017), 2) reduced excitatory input from the cardiovascular system to the rostral ventrolateral medulla (which regulates sympathetic activity) inhibits the sympathetic nervous system and lowers peripheral levels of cortisol (Mischel et al., 2015). Exercise training downregulates the number of AMPA (GluR3 subunit) receptors in the rostral ventrolateral medulla area (Subramanian et al., 2014), 3) increased cortisol levels induced by exercise influences central levels of endocannabinoids, which can reduce/inhibit excitatory inputs to the paraventricular nucleus and as a consequence inactivates the HPA axis and sympathetic activation (Ziegler et al., 2010), and 4) upregulation of β-endorphin (Yermal et al., 2010). The nuclei of neurons producing β-endorphin are located in the arcuate nuclei of hypothalamus, and their terminals are distributed throughout the CNS including the hypothalamic paraventricular nucleus which inhibits corticotropin-releasing hormone secretion (Sarkar and Zhang, 2013), and 5) increased galanin expression in the locus coeruleus, which suppresses the release of norepinephrine in the locus coeruleus (Voss et al., 2011; Lin and Kuo, 2013; Phillips and Fahimi, 2018). Experiments in animals (Murray et al., 2010) and humans (Legakis et al., 2000) demonstrate that exercise upregulates galanin expression in the locus coeruleus. Thus, exercise buffers the effects of stress from socio-psychological events such as the coronavirus pandemic and other behaviors causing anxiety (Brailovskaia et al., 2021).

Growth and neurotrophic factors have functional interactions, such that increases in one influence the release of the other in the brain (Deyama et al., 2019). Neurotrophic and growth factors mediate the beneficial effects of exercise on neurons, vascularization and neurotransmission (Basso and Suzuki, 2017; Delezie and Handschin, 2018). Thus, exercise training improves psychological, mood and mental health partly through the release of neurotrophic and growth factors (Cotman and Berchtold, 2002; Kandola et al., 2019). Central and peripheral levels of BDNF expression are lower in patients with MS and depression and this may be exacerbated during infection with COVID-19, as supported by findings that anti-depressant treatments and exercise training raise BDNF levels (White and Castellano, 2008; Silverman and Deuster, 2014). Exercise alters the levels of some monoamines (e.g., norepinephrine, serotonin, and tryptophan) to impact BDNF expression (Ivy et al., 2003; Phillips and Fahimi, 2018). In addition, endocannabinoids released from exercising muscles and neural terminals bind to their receptors (CB1 and CB2) to increase BDNF levels (Tantimonaco et al., 2014). BDNF increases the expression of tryptophan hydroxylase, which is involved in serotonin biosynthesis in neurons of the raphe nucleus (Siuciak et al., 1998; van Praag, 2009). BDNF increases synaptophysin and synaptobrevin levels which mediate neurotransmitter release (Kondo and Shimada, 2015); levels of synaptophysin and synaptobrevin are altered in cognitive disorders (Cotman and Engesser-Cesar, 2002). Exercise improves synaptic function by upregulating genes related to membrane trafficking, neurotransmitter vesicle recycling, synaptic plasticity (Bolton et al., 2000; Schinder and Poo, 2000). Thus, regular physical exercise reduces stress induced loss of BDNF levels and can alleviate depression, anxiety and improve mood (Marais et al., 2009; Szuhany and Otto, 2020). It is likely that neuroendocrine changes can dampen socio-psychological disorders associated with the coronavirus pandemic in patients with MS (Meeusen et al., 2001; Lin and Kuo, 2013; Pedersen and Saltin, 2015; Basso and Suzuki, 2017) (Figure 3).

Exercise promotes the release of endocannabinoids (Carek et al., 2011), which have roles in mood, analgesia, memory and reward systems (Basso and Suzuki, 2017). Plasma endocannabinoids are lipophilic and readily passes the BBB to interact with cannabinoid receptors in the brain. Eight days of voluntary exercise increased anandamide levels and the density and activity of hippocampal cannabinoid CB1 receptors (Hill et al., 2010), which are associated with neuronal plasticity in the brain (Tantimonaco et al., 2014). Binding to central or peripheral endocannabinoids receptors produces different responses (Raichlen et al., 2012), and can produce analgesia (Agarwal et al., 2007), stimulate reward centers in the brain to reduce anxiety and enhance euphoria (Dietrich and McDaniel, 2004), improve mood and lower depression by increasing the expression of BDNF (Tantimonaco et al., 2014), and finally modulate the release of dopamine (increased) and GABA (decreased) (Raichlen et al., 2012).

Exercise increases the levels of serotonin, dopamine and norepinephrine levels in the brain and spinal cord (Meeusen et al., 2001), and also increases the affinity of dopamine receptors (Lin and Kuo, 2013), decreases dopamine breakdown (Petzinger et al., 2007), promotes mRNA expression levels of dopamine D2 receptors; collectively, these changes reduce motor dysfunction (Vučcković et al., 2010), reduces loss of dopaminergic neurons induced by inflammation (Reiss et al., 2009), and increases dopamine synthesis (Lin and Kuo, 2013). The increased content of cerebral norepinephrine and dopamine induced by exercise are mediated by tyrosine hydroxylase, biosynthesizing enzyme common to the production of both neurotransmitters (Sutoo and Akiyama, 2003; Ma, 2008; Lin and Kuo, 2013). Norepinephrine regulates synaptic plasticity, increases neuronal survival and regeneration, and enhances mood (Ma, 2008; Basso and Suzuki, 2017).

Physical exercise increases the availability of tryptophan to stimulate the synthesis of serotonin in the brain (Chaouloff, 1989), while also modulating serotonin receptors (5-HT1A, 5-HT1B, and 5-HT2A) in anxiety and depression (Chennaoui et al., 2000; Guszkowska, 2004). Decreases in cerebral 5-HT1A and 5-HT2A receptors occur in patients with cognitive impairment such as MS (Meltzer et al., 1998). Stimulation of post-synaptic 5-HT1A and 5-HT3 receptors produces anti-depressant effects by upregulating hippocampal BDNF levels (Kondo et al., 2015; Kondo and Shimada, 2015).

Endogenous opioids such as β-endorphin, enkephalins, endorphins, and dynorphins are released from the anterior pituitary gland in response to exercise (Basso and Suzuki, 2017). In addition to increasing opioid peptides, exercise also modulates the binding affinity of endogenous opioids to mu (μ), kappa (κ) and delta (δ) receptors (Boecker et al., 2008). Exercise modulation of the sensitivity and number of opiate receptors (especially μ receptors) in the brain is associated with positive alterations in mood, depression, anxiety, analgesia, euphoria, and stress (Boecker et al., 2008; Dinas et al., 2011; Tantimonaco et al., 2014; Arida et al., 2015).

Collectively, regular physical exercise reduces the mental and psychological challenges associated with COVID-19 pandemic in patients with autoimmune diseases such as MS patients who are highly susceptible to mental and psychological issues. The positive changes induced by exercise training are mediated by alterations of neurotransmitters, neurotrophic factors, opioids, and their receptors.

## Exercise Training and Immunity

Chronic inflammation, which is associated with increased cytokine levels, is a pathologic hallmark of both MS and COVID-19 (Pedersen, 2009; Pedersen, 2017). Pro-inflammatory cytokines stimulate all aspects of acute phase responses, including the production of many acute phase proteins such as C-reactive protein (CRP) and IL-6 (Pedersen et al., 2001) and also upregulation of matrix metalloproteinases (MMPs) (Kurzepa et al., 2005).

Exercise produces anti-inflammatory effects through a variety of mechanisms that mitigate chronic inflammation in some diseases related to autoimmunity and hyper-inflammation (Figure 4) (Brandt and Pedersen, 2010; Gleeson et al., 2011). Plasma levels of pro-inflammatory cytokines (TNF-α, IL-1β, and IL-6) are increased during and after physical exercise (Pedersen et al., 2001), with increases in IL-6 greater than production other cytokines (Aral et al., 2014). Increases in IL-6 during exercise are due to activation of the SNS and activation of the HPA axis (Pedersen et al., 2001). Adipocytes, macrophages in adipose tissue, monocytes, brain, liver and exercising muscles are the primary sources of IL-6 during exercise (Gleeson et al., 2011; Coelho Junior et al., 2016). Muscle-derived IL-6 attenuates the production of some pro-inflammatory by cytokines (IL-1β, TNF-α) released by inflammatory cells and adipose tissue (Pedersen et al., 2001; Gleeson et al., 2011). Furthermore, IL-6 upregulates circulatory anti-inflammatory cytokines such as interleukin-1 receptor antagonist (IL-1ra), IL-10, and IL-4 (Pedersen et al., 2001; Suzuki et al., 2002; Steensberg et al., 2003; Pervaiz and Hoffman-Goetz, 2011). IL-1ra is mainly secreted by monocytes and macrophages and inhibits the pro-inflammatory functions of IL-1β (Freeman and Buchman, 2001), while IL-10 is mostly produced by Treg, Th1, Th2, and Th17 cells and also monocytes, macrophages, dendritic cells, B, and CD8^+^ T cells (Maynard and Weaver, 2008). IL-10 can downregulate the levels of MHC, intercellular adhesion molecule 1 (ICAM1), and costimulatory molecules (CD80 and CD86) on antigen-presenting cells (APCs) (Maynard and Weaver, 2008). Inhibition of some pro-inflammatory cytokines and adaptive immune components is another role of IL-10 in mitigating the capacity of effector T cells to maintain inflammatory responses (Moore et al., 2001; Maynard and Weaver, 2008). IL-6 secreted from exercising muscles augments cortisol release (which is an anti-inflammatory agent) (Steensberg et al., 2003).

Increased vagal tone occurs during regular physical exercise and after some adaptations to exercise (Rodrigues et al., 2014). The parasympathetic nervous system activates the cholinergic anti-inflammatory pathway, consisting of vagal afferents, motor and efferent projections (Bonaz et al., 2016). Afferent projections deliver the information on peripheral immune conditions to the CNS (Pavlov and Tracey, 2012). Increased cholinergic anti-inflammatory pathway activation promotes macrophage transformation from M1 to M2 subtypes; the M2 phenotype produces anti-inflammatory cytokines and T regulatory cells (Tregs) which nave roles in immune suppression (Rocha et al., 2016). On the other hand, acetylcholine released from efferent outflows activates nicotinic receptor α7 on immune cells to prevent further release of pro-inflammatory cytokines (Pavlov et al., 2006).

Patients with MS and COVID-19 are often forced to adopt sedentary lifestyle, which leads to an accumulation of visceral fat, which is strongly associated with the infiltration of pro-inflammatory cytokines, macrophages, T cells and the appearance of chronic systemic low-grade inflammation (Ouchi et al., 2011) as well as the migration of peripheral blood mononuclear cells to adipose tissue (Gautier et al., 2009; Zeyda et al., 2011). Increases in the size of adipocytes and the number of infiltrated immune cells stimulate the recruitment of macrophages to adipocytes that is mediated by chemokine ligand 2 and 3 (CCL2, CCL3) (also known as MIP1α) (Bruun et al., 2005). Immune cells in adipocytes release chemokines that mediate upregulation of complimentary chemokine receptors on peripheral blood mononuclear cells (Gleeson et al., 2011). Exercise inhibits the migration of peripheral blood mononuclear cells to inflamed adipose tissue (Kawanishi et al., 2013) through the secretion of chemokines from other sources and thereby causes an internalization of chemokine receptors on peripheral blood mononuclear cells (Maffei et al., 2009). Further, exercise reduces the release of chemokines from adipose tissue, which attenuates the infiltration of macrophages (Kanda et al., 2006). Thus, exercise reduces the migration of peripheral blood mononuclear cells to adipose tissues and reduces inflammation by expediting the phenotypic conversion of macrophages from M1 (pro-inflammatory type) to M2 (anti-inflammatory) (Kawanishi et al., 2010).

Exercise reduces the expression of tissue ICAM1, which regulates the docking of inflammatory cells on the endothelium, extracellular matrix, epithelium, and mediates the interaction between T cells and target cells; hence, regular exercise inhibits macrophage infiltration into adipose tissues (Zoppini et al., 2006; Kawanishi et al., 2010). Exercise-induced reductions in circulatory inflammation also occur by modulating monocytes (Gleeson et al., 2006). Toll-like receptors are transmembrane proteins that are highly expressed on monocytes, where they have important roles in the diagnosis of microbial pathogens and tissue damage (Kaisho and Akira, 2006). Activation of Toll-like receptors produces pro-inflammatory cytokines (Takeda et al., 2003). Trained individuals have reduced expression levels of Toll-like receptors (TLR1, 2, and 4) in their monocytes and lower release of inflammatory cytokines (Stewart et al., 2005; Flynn and McFarlin, 2006; Gleeson et al., 2006; Oliveira and Gleeson, 2010). In addition, regular physical exercise attenuates the number of circulating inflammatory monocytes (CD14^low^ CD16^+^), which are rich in Toll-like receptors (TLR4) on their surfaces (Skinner et al., 2005; Timmerman et al., 2008; Simpson et al., 2009). Exercise reduces visceral fat mass and lowers the production of pro-inflammatory adipokines (TNF-α, leptin, retinol binding protein 4), lipocalin 2, IL-18, chemokine ligand 2 (CCL2 or MCP-1), CXC-chemokine ligand 5, angiopoietin-like 2 to create an anti-inflammatory environment (Petersen and Pedersen, 2005; Lim et al., 2008; Mathur and Pedersen, 2008; Mujumdar et al., 2011).

Reduced levels of Treg cells leads to autoimmunity and stimulates immune responses to exogenous antigens (Fernandez et al., 2008; Paust et al., 2011). These cells can suppress immune responses by producing forkhead box P3 proteins (Sakaguchi, 2005). Thus, increasing the numbers of circulatory Treg cells, for example by exercise, can limit inflammation in diseases such as MS (Yeh et al., 2006; Duffy et al., 2019; Goverman, 2021). In response to antigen stimulation, Treg cells causes the release of anti-inflammatory cytokines (IL-10, TGF-β) and the change of T helper 1 (Th1) cells (pro-inflammatory phenotype) to anti-inflammatory Th2 cells by increasing forkhead box P3 proteins (Yeh et al., 2009).

Peripheral and central levels of pro-inflammatory cytokines including TNF-α, IFN-ϒ, IL-6, and IL-1β are increased in neurological diseases (Mee-Inta et al., 2019). Activation of microglia by pro-inflammatory cytokines results in microgliosis which is then followed by the production of large amounts of IL-1β (Mee-Inta et al., 2019). Increase in IL-1β mediated by microglia exacerbates inflammation in the CNS by reactivating infiltrated lymphocytes and also by incurring astrogliosis (Pekny and Pekna, 2014). Exercise suppresses microgliosis and reduces inflammation by increasing the levels of anti-inflammatory cytokines (Mee-Inta et al., 2019).

## Exercise Training and BBB Permeability

Regular exercise alters BBB permeability by causing changes in tight-junction proteins (occludin, claudins, and zonula occludens (ZOs)) and in supporting astroglial cells (Figure 5). There is insufficient evidence that exercise alters the function of these proteins in MS, although a recent report indicates that endurance exercise after EAE induction (animal model of MS) increases claudin-4 and occluding levels (Souza et al., 2017). Some clinical evidence suggests that exercise affects BBB permeability by increasing oxidative capacity and reducing inflammation (Souza et al., 2017; Chupel et al., 2018). Activation of matrix metalloproteinases (MMPs) produced by reactivated astrocytes and invasive T lymphocytes can disrupt cerebrovascular base-membrane and endothelial tight junction proteins in inflammatory diseases such as MS (Rempe et al., 2016). Exercise training modulates the concentrations of permeable BBB markers such as MMPs and S100β in MS patients (Zimmer et al., 2018; Negaresh et al., 2019). The effects of exercise on BBB changes in nonclinical conditions were monitored using peripheral markers such as serum levels of S100β (*S100 calcium-binding protein B, a protein expressed by mature astrocytes*) were used to monitor changes in BBB permeability (Koh and Lee, 2014). Exercise-induced changes in BBB permeability is related to hyperthermia, increases in circulatory concentrations of ammonia, adrenaline, noradrenaline, inflammatory mediators, central neurotransmitters (serotonin and glutamate), ROS production, and growth factors (Watson et al., 2006; Nierwińska et al., 2008; Małkiewicz et al., 2019; Suzuki et al., 2020). Increased lactate produced during exercise triggers the expression of hypoxia-inducible factor 1-alpha (HIF1-α) followed by the activation of vascular endothelial growth factor (VEGF)-A expression in astrocytes (Zimmer et al., 2019) and disruption of the tight-junction proteins (claudin-5 and occluding) to alter BBB permeability (Brambilla et al., 2005). Thus, exercise in nonclinical conditions increases BBB permeability to meet the increased neural demands caused by physical exercise.

Astrocytes interact with tight-junction proteins to maintain BBB integrity (Razi et al., 2022a). Astrocytes are glial cells having extensive connections with adjacent cells such as endothelial cells. Inflammatory conditions cause these glial cells to undergo astrogliosis that is characterized by morphological and functional changes with upregulation of GFAP (Abbott et al., 2006; Liddelow and Barres, 2017). Glutamate excitotoxicity, oxidative stress and pro-inflammatory cytokines (IL-6, IL-1β, and TNF-α) trigger astrogliosis and increases in BBB permeability in diseases such as MS and COVID-19 (Burda and Sofroniew, 2014). Thus, reducing reactive astrogliosis by downregulation of GFAP can be a therapeutic target for diseases involving disorders of BBB permeability (Figure 5) (Alvarez et al., 2013), although there is limited evidence to support this, especially in diseases involving inflammatory conditions (Razi et al., 2022a). Six-weeks of exercise training in an animal model of MS reduced both GFAP expression and astrogliosis (Mandolesi et al., 2019). Some potential mechanisms for exercise-induced downregulation of GFAP include: 1) reduced levels of cytokines released by activated microglia and astrocytes, 2) upregulation of Tregs in the CNS and phenotype alterations of Th1 to Tregs, 3) suppression of ROS production and oxidative stress, and 4) inhibition of microglial activation (Santin et al., 2011; Bernardi et al., 2013; Radak et al., 2016; Gentile et al., 2019; Quan et al., 2020) by increased secretion/expression of anti-inflammatory cytokines (IL-1ra, IL-10, and IL-4) and upregulation of the CD200 and CD200R glycoproteins on neurons and microglial cells, respectively (Mee-Inta et al., 2019). Inhibition of microglial activation in autoimmune diseases reduces the release of microglia-mediated pro-inflammatory cytokines such as IL-1β and consequent reduction of astrocyte activation (Mee-Inta et al., 2019). Exercise-induced cortisol release downregulates the expression of GFAP by astrocytes (Bernardi et al., 2013). Exercise-induced angiogenesis, reportedly, promotes astrocyte proliferation to strengthen the neurovascular unit (NVU) and preserve the integrity of the BBB (Li et al., 2005).

In summary, regular exercise improves BBB integrity in some neurological conditions by mitigating inflammatory states, increasing tight-junction proteins, promoting angiogenesis, and favouring astrogliocytosis (astrocyte proliferation) over astrogliosis.

## Exercise Training and Plaque Reduction Through Remyelination

Myelin is required for the conduction of neural impulses, and demyelination of neurons and white matter atrophy is a characteristic of MS (Bando, 2020). Oligodendrocytes and oligodendrocyte precursor cells are susceptible to damage by inflammatory mediators, ROS, and oxidative stress in MS (Yoon et al., 2016; Feter et al., 2018). Damaged oligodendrocytes and demyelination lead to the neurological deficits (Cheng J. et al., 2020). Oligodendrocyte precursor cells are quiescent cells that can migrate to demyelinated areas to restore myelin (Jiang et al., 2017; Jensen et al., 2018). Physical exercise can affect the remyelination process by influencing the neuronal microenvironment in the CNS (Figure 6) and by a variety of related mechanisms including increased upregulation of neurotrophic and growth factors such as BDNF, insulin-like growth factor-1 (IGF-1), neurotrophin-3 (NT-3), and their receptors [tyrosine kinase receptor B (TrkB) and IGF-1R] to influence neural precursor cells and oligodendrocyte precursor cells to proliferate and differentiate to oligodendrocyte precursor cells and oligodendrocytes, respectively (Gallo and Armstrong, 2008; Ahn et al., 2016; Jensen and Yong, 2016; Tomlinson et al., 2016; Feter et al., 2018). These neurotrophic and growth factors influence myelin production by stimulating the phosphoinositide 3-kinases (PI3K)-Akt-mTOR pathway (Carson et al., 1993; Cao et al., 2003; Lin et al., 2005).

Exercise increases silent mating type information training (SIRT1) levels (Sarga et al., 2013), which upregulates peroxisome proliferator-activated receptor gamma (PPAR-γ) coactivator 1-alpha (PGC-1α), a transcriptional factor for mitochondrial biogenesis (Cheng J. et al., 2020). Increases in mitochondria reduces ROS-derived oxidative stress and protects myelin from oxidative damage in MS (Rafalski et al., 2013; Ng et al., 2015). Physical exercise lowers levels of 4-hydroxynonenan (4-HNE, a marker of lipid peroxidation) in the brain (Yoon et al., 2016). Importantly, improved mitochondrial function due to exercise training increases the activity of acetyl-CoA carboxylase 1 and 2, enzymes that provide malonyl-CoA for synthetizing long-chain fatty acids required for remyelination (Sedel et al., 2015; Sedel et al., 2016). Furthermore, increased exercise-induced PGC-1α reduces demyelination by increasing antioxidant enzyme levels to offer greater protection from oxidative stress (St-Pierre et al., 2006), while also promoting remyelination by modulating lipid production, oligodendrocyte differentiation and myelin proteins such as myelin basic protein and proteolipid protein (Camacho et al., 2013; De Nuccio et al., 2015).

Regular physical exercise inactivates microglia and also modulates their phenotype conversion from M1 (inflammatory) to M2 (neuroprotective) (Kohman et al., 2012; Franco and Fernandez-Suarez, 2015). M1 microglia cause oligodendrocyte apoptosis and suppress remyelination by increasing antigen presentation and producing toxic cytokines, while M2 microglia have neurotrophic effects by mitigating local inflammation, clearing oligodendrocyte debris and releasing neurotrophic factors (Miron et al., 2013). Exercise leads to an upregulation of the fractalkine receptor proteins (*CX3CL1/CX3CR1; mediators of chemotaxis and adhesion of immune cells*) to polarize microglia to a neuroprotective phenotype (IGF1/Iba1 positive microglia), and increase their phagocytic activity to expedite the clearance of myelin debris (Vukovic et al., 2012; Ransohoff and El Khoury, 2016). M2 microglia trigger the differentiation of oligodendrocyte precursor cells and attenuate apoptosis of newborn oligodendrocytes as important components of remyelination (Miron et al., 2013). Additional mechanisms for exercise-induced remyelination include increases in the density of remyelinated axons, and a restored g-ratio (*g-ratio measures myelin thickness and is calculated by dividing the inner axon dimeter by the outer myelin diameter*) (Feter et al., 2018; Jensen et al., 2018).

## Practical Considerations

Survivors of acute viral respiratory diseases such as COVID-19 endure neuropsychological deficits and a poor quality of life (QOL) that can last for 1 year or more (Yeo, 2020; Gentil et al., 2021). Patients can experience muscle weakness and atrophy, tendon, and neuromuscular impairments in intensive and long-term health care (Gentil et al., 2021). Engaging in regular physical exercise prior to infection (and even early after infection) can reduce these complications (Calverley et al., 2020; Hekmatikar et al., 2021) and also limit mental and physical stress (Silverman and Deuster, 2014). The beneficial adaptions to exercise training can occur within 4 days to 26 weeks (Batouli and Saba, 2017). The World Health Organization (WHO) recommends individuals undertake at least 150 min exercise with moderate-intensity or 75 min with high-intensity exercise per week (Norum, 2005).

Viral infection in MS patients is often associated with an increased risk of relapsing (De Keyser et al., 1998). Patients with MS are sensitive to increases in body temperature during exercise sessions (Razi et al., 2022b), and a supervised muscle strengthening training program should be modified according to the stage of the disease (Smith et al., 2006; Pedersen and Saltin, 2015). Physical exercise is not recommended during any systemic viral disease, since inflammatory reactions in muscle cells and coronary artery walls increase the risk of cardiac sudden death during infection (Inciardi et al., 2020). The average time from initiation to clinical recovery from COVID-19 infection is 2 weeks (Barker-Davies et al., 2020) and this period can last from 3 to 6 weeks for patients with severe clinical disease (Woods et al., 2020).

Inclusion of the resistance training in daily activities for at least two sessions per week expedites recovery from infection (Norum, 2005). The resistance training program recommended amid the COVID-19 pandemic involves a low number of repetition (≤6 repetition) and a long periods of rest between sets (≥3 min) (Gentil et al., 2021). This training protocol is suitable for patients with MS who are also infected with the SARS-CoV-2 virus, since patients with MS are sensitive to hyperthermia and an additional respiratory infection can further limit participation in aerobic exercise (Albesa-Albiol et al., 2019; Garnacho-Castaño et al., 2021). Resistance training can improve mood, and limit states of depression and anxiety (Gordon et al., 2017; Gordon et al., 2018), while progressive strength training for 12 weeks in MS patients is the best strategy to promote muscle strength and improve depression, fatigue, and QOL (Dalgas et al., 2009; 2010).

Exercise also improves immunological responses in MS and COVID-19 (Gentile et al., 2019; da Silveira et al., 2021). Moderate level of exercise improves the ability of the immune system to limit viral infections (Nieman et al., 2011). Vigorous exercise improves the anti-inflammatory actions of IL-10 (Wang et al., 2012). High-intensity exercise improves neuronal conduction velocity (van Meeteren et al., 1997), while moderate and resistance aerobic exercise increases remyelination of axons (Bobinski et al., 2011). The beneficial effects of exercise on body tissues, especially the brain, are mediated through neurotrophic factors (Tari et al., 2019). Moderate level of exercise increases serum levels of neurotrophic factors in patients with MS and also in healthy individuals (Gold et al., 2003). Thus, physical exercise with moderate intensity can improve brain health of patients with MS during and after the COVID-19 pandemic.

## Conclusion

Regular physical exercise mitigates mental and psychological disorders associated with COVID-19 infections in patients with MS by causing changes in neurotransmitters, neuromodulators, opioids, and neurotrophic and growth factors. Regular exercise leads to positive changes in central and peripheral immune systems and induces an anti-inflammatory milieu to limit the effects of the cytokine storm associated with MS and COVID-19. Thus, regular exercise training has pronounced central and peripheral effects that can be used as prophylactic and reparative interventions to improve brain health.

## Abbreviations

4-HNE, 4-hydroxynonenan; ACE2, Angiotensin-converting enzyme 2; BBB, Blood-brain barrier; BDNF, Brain-derived neurotrophic factor; CB1, Cannabinoid type 1 receptor; CNS, Central nervous system; COVID-19, Coronavirus disease-19; CREB, cAMP response element-binding protein; CRP, C-reactive protein; GFAP, Glial fibrillary acidic protein; HCoVs, Human coronaviruses; HPA, Hypothalamic-pituitary-adrenal; IFN-ϒ, Interferon-gamma; IL-1ra, Interleukin-1 receptor antagonist; MERS-CoV, Middle east respiratory syndrome-related coronavirus; MMPs, Matrix metalloproteinases; MS, Multiple sclerosis; NMDA, N-methyl-D-aspartate receptor; Pgc-1α, Peroxisome proliferator-activated receptor gamma (PPAR-γ) coactivator 1-alpha; ROS, Reactive oxygen species; SIRT1, Silent mating type information training; TNF-α, Tumor necrosis factor-alpha; Tregs, T regulatory cells; VEGF, Vascular endothelial growth factor; WHO, World health organization.
